# “The Rush Video‐Based Tic Rating Scale‐Revised: A Practice‐Oriented Revision”

**DOI:** 10.1002/mdc3.13922

**Published:** 2023-11-27

**Authors:** Leonie Felicia Becker, Tina Rawish, Tobias Bäumer, Christian Beste, Veit Roessner, Alexander Münchau, Julius Verrel

**Affiliations:** ^1^ Institute of Systems Motor Science, University of Lübeck Lübeck Germany; ^2^ Department of Pediatrics University Hospital Medical Center Schleswig‐Holstein Lübeck Germany; ^3^ Cognitive Neurophysiology, Department of Child and Adolescent Psychiatry, Faculty of Medicine TU Dresden Dresden Germany; ^4^ University Neuropsychology Centre, Faculty of Medicine, TU Dresden Dresden Germany; ^5^ Cognitive Psychology, Faculty of Psychology Shandong Normal University Jinan China

**Keywords:** Tourette, modified rush video‐based tic rating scale, tic, video based rating

We appreciate the effort of Riechmann et al.^1^ to standardize and improve the Modified Rush Video‐Based Tic Rating Scale (MRVS).^2^ However, we consider some of the proposed changes as problematic and their evaluation as partially flawed, as detailed below.

Capturing representative tics in an artificial observational situation is facilitated by an explicit instruction not to voluntarily suppress tics, but such an instruction is missing. In fact, the proposed instruction to “keep eyes open” is unfortunate, as it will likely result in suppression of blinks/blink tics. The proposed reduction of rating time likely leads to an underrepresentation of tics, particularly in patients with milder symptoms, resulting in the potential loss of quantitative and qualitative information.^3^ This is particularly evident for typical bouts of tics, occuring after the experimenter left the room. Instead the authors recommend not evaluating the first minute of this video segment. Moreover, the authors claim that previous research indicated “high interrater reliability, fair correlations to clinician‐based rating scales, and sufficient sensitivity to change”^1^ for tic count measures based on 1‐ to 5‐minute rating durations, whereas the cited reference explicitly recommends using at least 5‐minute intervals.^4^

The authors claim that “reducing the rated recording time from 2 to 1 min does not influence motor and phonic tic counts”.^1^ However, their comparison of tic counts from a single versus two 1‐minute intervals is unconvincing for multiple reasons. First, the original MRVS procedure entails rating two 2.5‐minute (not 1‐minute) intervals. Second, the shared variance for motor tic frequency is rather low for measures meant to capture identical phenomena. Third, the effect of reducing the rating interval is not reported for the other items or the total score. Fourth, the analysis only involves a small, non‐representative sub‐sample (n = 20).

Extending the range covered by tic frequency categories makes sense, but the proposed bin widths appear arbitrary and we recommend using fixed bin widths (e.g., 25). The reported increase in internal consistency for the revised score is at least partially explained by increasing the number of items (from 6 to 9) and by introducing presumably correlated items (complexity, intensity, and number of tic types). Statistical significance of numerical differences is not reported. Importantly, a high internal consistency is not required for scales measuring heterogeneous phenomena.

Contrary to the authors' claim, a high convergent validity between Yale Global Tic Severity Scale (YGTSS) and MRVS is not desirable, as both assess complementary aspects of a complex and variable clinical condition, based on different sources of information and at different time scales. This is evident, for example, in the fact that the strength of stimulus–response binding correlated with video‐based assessments of tic frequency using MRVS,^5^ but not with YGTSS. Additionally, the convergent validity (with YGTSS) is evaluated only on a small subsample (n = 40), and the statistical significance of the numerical differences is not reported. Furthermore, a (numerically) substantially higher correlation (*r* = 0.77) with the total tic score in the original MRVS has been reported.^2^

Taken together, we recommend reconsidering and more thoroughly evaluating the suggested changes.

## Author Roles

(1) Manuscript Preparation: A. Writing of the First Draft, B. Review and Critique.

L.F.B.: 1A, 1B

T.R.: 1A, 1B

T.B.: 1B

C.B.: 1B

V.R.: 1B

A.M.: 1A, 1B

J.V.: 1A, 1B

## Disclosures

**Ethical Compliance Statement:** The authors confirm that the approval of an institutional review board was not required for this work. Informed patient consent was not necessary for this work. We confirm that we have read the Journal's position on issues involved in ethical publication and affirm that this work is consistent with those guidelines.

**Funding Sources and Conflict of Interest:** This work was supported by the Deutsche Forschungsgemeinschaft (DFG, FOR 2698). The authors declare that there are no conflicts of interest relevant to this work.

**Financial Disclosures for the previous 12 months:** L.F.B. is employed by the University Medical Center Schleswig‐Holstein, Campus Lübeck. T.R. is employed by the University Medical Center Schleswig‐Holstein, Campus Lübeck. T.B. has provided consultancies to Pelzerhaken Children's Center; is on Advisory Boards of Allergan/AbbVie, Ipsen Pharma, and Merz Therapeutics; has received honoraria from Allergan/AbbVie, Ipsen Pharma, and Merz Therapeutics; has received grants from Allergan/AbbVie, Ipsen Pharma, and Merz Therapeutics; and is employed by the University of Lübeck and the University Medical Center Schleswig‐Holstein, Campus Lübeck. C.B. has stock ownership in medically related fields in Bayer, Genzyme, Novartis, Teva, and GlaxoSmithKline; has partnerships in Bayer, Genzyme, Novartis, Teva, and GlaxoSmithKline; has received honoraria and support from foundations Friede Springer Stiftung, Else Kröner Fresenius Stiftung, and CHDI; has received academic research support from Deutsche Forschungsgemeinschaft (DFG), SFB 940, SFB TRR 265, and FOR 2698; and is employed by the TU Dresden, Germany. V.R. is on advisory boards of German Tourette Society, German Society of Obsessive‐Compulsive Disorder; has received honoraria from Hogrefe; has received grants from the Federal Ministry of Education and Research, the Innovation Fund of the Federal Joint Committee, the German Research Foundation, MSE Pharma, and Takeda; is employed by the Department of Child and Adolescent Psychiatry and Psychotherapy, and Faculty of Medicine Carl Gustav Carus of the Technische Universität Dresden. A.M. has provided consultancies for Desitin, Admedicum, PTC Therapeutics, Novartis, and Barmer; is on advisory boards of German Tourette syndrome Association, Alliance of patients with chronic rare diseases, and Novartis; has received honoraria for lectures from GlaxoSmithKline, Desitin, Teva, and Takeda; has received grants and commercial research support from Pharm Allergan, Ipsen, Merz Pharmaceuticals, and Actelion; has received academic research support from Deutsche Forschungsgemeinschaft (DFG) on projects 1692/3–1, 4–1, SFB 936, FOR 2698 (project numbers 396, 914, 663, 396, 577, 296, 396, 474, 989) and European Reference Network‐Rare Neurological Diseases (ERN‐RND; Project ID No 739510); has received support from Foundations of Tourette Syndrome Association (Germany), Interessenverband Tourette Syndrom (Germany), and CHDI; is employed by the University of Lübeck and University Medical Center Schleswig‐Holstein, Campus Lübeck; has received royalties for the book Neurogenetics (Oxford University Press). J.V. is employed by the University Medical Center Schleswig‐Holstein, Campus Lübeck.
